# Factors associated with mothers’ health care-seeking behaviours for childhood fever in Burkina Faso: findings from repeated cross-sectional household surveys

**DOI:** 10.1186/s41256-022-00270-2

**Published:** 2022-10-20

**Authors:** Hermann Badolo, Aristide R. Bado, Hervé Hien, Nicolas Méda, A. Sathiya Susuman

**Affiliations:** 1 Department of Statistics and Population Studies, Faculty of Natural Sciences, University of the Western Cape, Cape Town, South Africa; 2 National Institute of Public Health (INSP), Ouagadougou, Burkina Faso; 3 Health Sciences Research Institute (IRSS), Ouagadougou, Burkina Faso; 4 Health Sciences Research Institute (IRSS), Bobo-Dioulasso, Burkina Faso; 5 West African Health Organization (WAHO), Bobo-Dioulasso, Burkina Faso; 6 Health Sciences Training and Research Unit, Université Ouaga I Pr. Joseph Ky-Zerbo, Ouagadougou, Burkina Faso

**Keywords:** Childhood fever, Illness, Prevalence, Health care, Health care-seeking, Burkina Faso

## Abstract

**Introduction:**

Fever is one of the most frequent reasons for paediatric consultations in Burkina Faso, but health care-seeking behaviours and the factors associated with health care-seeking in the event of childhood fever are poorly documented. This study aims to analyse the health care-seeking behaviours and the factors associated with health care-seeking for childhood fever in Burkina Faso.

**Methods:**

This study used the data from the baseline and endline surveys conducted to evaluate the impact of the Performance-Based Financing program in Burkina Faso. Univariate and multivariate binary logistic regression analyses were used to identify the factors associated with appropriate healthcare-seeking for childhood fever. Odds ratios were estimated to assess the strength of associations and 95% confidence intervals (CIs) were used for significance tests. Data were cleaned, coded and analysed using Stata software version 16.1.

**Results:**

Among the children under five who had a fever, 75.19% and 79.76% sought appropriate health care in 2013 and 2017, respectively. Being 24–59 months old (AOR: 0.344, 95% CI 0.182–0.649 in 2013 and AOR: 0. 208, 95% CI 0.115–0.376 in 2017), living in a very wealthy household (AOR: 2.014, 95% CI 1.149–3.531 in 2013 and AOR: 2.165, 95% CI 1.223–3.834 in 2017), having a mother with a secondary or higher level of education or having made at least four antenatal care visits were significantly associated with seeking appropriate health care for childhood fever. Living in an area where the health facility is safe was also significantly associated with seeking appropriate care for childhood fevers.

**Conclusions:**

The findings underscore the need to concentrate efforts aiming at sensitizing the population (especially women of childbearing age) to improve sanitation and the use of family planning (household composition), skilled antenatal care and postnatal care to help reduce the prevalence of fever in children under five and improve the use of medical healthcare for childhood fever.

## Introduction

Each year, some 8 million children in developing countries die before they reach their fifth birthday, and many die in the first year of life [1]. Eight in ten of these deaths are due to neonatal conditions, diarrhoea, acute respiratory infection, fevers alone or those associated with other symptoms often overlapping with signs of malaria in particular [1–7]. Most deaths due to diarrhea, fever, cough, pneumonia, and malaria in young children from developing countries could be prevented by relatively simple treatments and interventions [1, 8].

Fever as a perception of high body temperature is often viewed by parents as an illness itself rather than a symptom or sign of illness [6, 9]. It is one of the most frequent reasons for paediatric consultations [10], accounting for nearly 30% of paediatric consultations worldwide. Fevers, which very often mask other symptoms of malaria, have always been one of the leading causes of morbidity and mortality in childhood (40% of deaths under five) [11]. In addition, several studies have reported that children who accumulate a nutrient deficit in the neonatal period have their risk of death increased during the neonatal period. This disadvantage persists for the first five or more years of life [12–14].

In Burkina Faso, Health service delivery is organized in a three-tier system, with primary facilities, located in rural areas; district hospitals located in each district capital; and regional and national referral hospitals located in the regional capitals and in the national capital Ouagadougou [15]. Public facilities provide the vast majority of health services [15]. The data on the prevalence of fever are not encouraging. The country has a high prevalence of malarial infection and fever among children under five. Indeed, according to the malaria indicators’ survey (MIS) in Burkina-Faso, carried out by the National Institute of Statistics and Demography (INSD) in 2014, four out of ten children (40%) had fever during the two weeks preceding the survey [16]. According to the same survey, in 46% of children under five who had a fever in the two weeks preceding the survey, the fever was not treated, and only 35% of children who had a fever took antimalarial drugs [16]. Burkina Faso is one of the ten countries with the highest number of malaria cases and associated deaths (3% of cases and deaths worldwide). Malaria is responsible for 43% of health facility use and 22% of deaths [17].

Despite this burden, one of observations regarding health care-seeking is the long-time taken by parents before seeking health care in health facilities. The cases generally encountered in health facilities are therefore serious cases that have been the subject of many treatment attempts with other therapists outside the national health system [18–20]. Evidence suggests that the factors associated with the use of health services in the event of children’s illness are both multiple and complex, relating to various fields and exerting their influences at the individual, household, community and national levels [21, 22]. In Burkina Faso and elsewhere, previous studies conducted documented those factors such as parents' (especially mothers') sense of competence for detecting signs of illness [23, 24], the distance between home and the health facility, long periods of waiting for medical services and direct payment for care were the main reasons for low healthcare utilization in developing countries [19, 25–29].

However, it is recognized that the early and adequate management of fever considerably reduces the incidence of severe cases [24, 30–32]. Nevertheless, little is known about the differential prevalence of childhood fever and the factors associated with health-seeking behaviours by mothers for fever in their children in Burkina Faso. In Burkina Faso, apart from the results of demographic and health surveys (DHS) and apart from the malaria indicators’ survey, which makes a distribution of the prevalence of fever according to certain socio-demographic characteristics [16, 33], the differential prevalence of this childhood illness, healthcare-seeking behaviours and the factors associated with health care-seeking in the event of children’s fever are poorly documented.

Regarding the public health problem posed by fever, the analysis of the differential prevalence of infantile fever, care-seeking behaviours and factors associated with the mothers seeking care in the event of infantile fever in Burkina Faso are all needed in order to generate knowledge that can inform program planners and policymakers working in the field of child health. This study aims to analyse the health care-seeking behaviours and the factors associated with health care-seeking for childhood fever in Burkina Faso.

## Methods

### Setting

The preliminary results of the 5th general population and housing census of Burkina Faso, carried out in 2019, indicated a total resident population of 20,487,979 inhabitants [34]. According to the same source, the vast majority of the population (73.7%) lives in rural areas in 2019 [34]. Moreover, according to the results of the demographic module of the continuous multisectoral survey carried out in 2019 in Burkina Faso, the vast majority of the population was affected by illiteracy (65.5% in 2014), and the poverty coverage rate at the national poverty line was estimated at 40.10% of the total population [35]. The 2018 Human Development Index of the United Nations Development Program ranks Burkina Faso 182nd out of 189 countries and territories with comparable data. According to the 2010 demography and health survey results for Burkina Faso, less than 6 in 10 women (58.4%) make 2 to 3 antenatal visits during pregnancy, and only 3 in 10 women (33.1%) make 4 or more visits during pregnancy [33].

### Study design

**Fig. 1:**
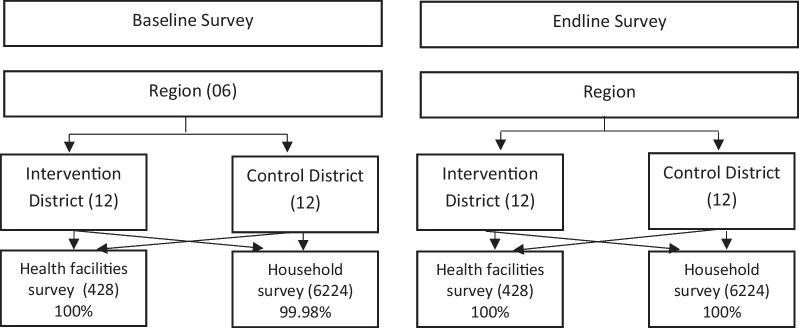
Survey design diagram

The study was conducted in six administrative regions (Boucle du Mouhoun, Centre-Est, Centre-Nord, Centre-Ouest, Nord and Sud-Ouest) of Burkina Faso. These six regions were chosen on the basis of the level of their health indicators. Of the six target regions, four have health indicators below the national median. Within each region, two health districts were selected by the government to receive the intervention based on poor results on four selected indicators: contraceptive prevalence rate, assisted deliveries, antenatal consultations and postnatal consultations [36] (Fig. 1).

Two study populations were used in this study. First, all permanent female residents of the study area who were pregnant or gave birth at least once in the last two years preceding the baseline and endline survey, irrespective of the outcome of delivery. Women of childbearing age who did not give birth at least once in the past two years prior to this survey and/or residing in the study area for less than six months were excluded from this study. Second, the target population for this study was all children aged 0–59 months who had a fever or not in the four weeks preceding the survey and their mothers.

### Data

To achieve the objectives of this study, two quantitative data sources were used: baseline (2013) and endline (2017) survey data for the impact evaluation of Performance-Based Financing (PBF) in Burkina Faso. The PBF impact assessment was a blocked-by-region cluster random trial based on a pre-post comparison design. In the protocol, it was planned to trace households and health facilities from the baseline survey to the final survey. The sample was derived in a three‐stage cluster sampling procedure, described in detail elsewhere [36].

Data from the baseline and endline surveys for the impact assessment of PBF in Burkina Faso was used for this study. The baseline and endline surveys collected data on household characteristics and household members, the health status of each household member and the use of health services, perception of the quality of services, antenatal care, postnatal care, immunization of children and use of the services of community health workers. This survey also provides information on the evaluation of the health facility, exit interviews after the consultations for children under five and for women seen in antenatal care and the distance between the home and the health facilities. Data was collected during the malaria season only.

### Variables

#### Outcome variables

In this study, there were two dependent variables. The first was constructed from self-reported information collected in face-to-face interviews. Reports of fever were classified into two categories: those reported to have had a fever in the last four weeks preceding the survey and those not reported to have a fever in the last four weeks preceding the survey. This outcome variable, therefore, includes two modalities: presence of fever = 1 and absence of fever = 0.

The second variable was appropriate healthcare-seeking. Seeking appropriate healthcare has been defined as seeking healthcare from all public or private health facilities, private doctors and community health workers but excluding non-medical care, pharmacies, shops and traditional healers [37].

These two outcome variables were based on self-reported information collected in face-to-face interviews.

#### Explanatory variables

The description of the differential prevalence of fever in children under five was made, not only with the classic variables (sex of the child, birth order, place of residence, region of residence, mothers’ level of education, wealth status, etc.) [21], widely studied by DHS and MIS, but with additional variables such as household composition, child immunization status, antenatal and postnatal care, the methods of garbage disposal, the sources of drinking water and the method of evacuating human excreta [38].

Regarding the analysis of the factors associated with appropriate health care-seeking behaviours, several groups of variables were considered. The variables describing the socio-demographic characteristics of the child, their mother and their household are as follows: child's sex, child's age, child's vaccination status, mother's age, mother's marital status, mother's level of education, mother's occupation, mother’s use of antenatal and postnatal care, and wealth status. The group of variables describing the morphological characteristics of the household includes the following: the size of the household, the number of children under five and the presence of elderly persons. The group of variables characterizing the health facilities includes the waiting time before receiving care and the safety and confidence in the health facility. The following questions were asked for each variable characterizing the health facilities:

- Waiting time: During the last visit, how long did you/[NAME] have to wait before seeing a health worker?
- Safety: The level of security in the area does not allow people in the community to use the health services available ? With the following terms: agree, neither agree nor disagree, Disagree
- Confidence: You have complete confidence in the decisions of the healthcare personnel regarding medical treatment at this healthcare facility ? With the following terms: agree, Neither agree nor disagree, Disagree

All these variables were self-reported.

### Statistical analysis

Several statistical methods were used to answer the research questions. The analytical strategy is based on two successive stages that are linked together to provide additional knowledge. The first analytical phase consists mainly of a description of the differential prevalence of fever in children under five years of age. To describe the differential prevalence of fever, cross-tables and their statistical associations were used (95%).

Univariate and multivariate binary logistic regression analyses were used to identify factors associated with appropriate health care-seeking behaviours for childhood fever. Odds ratios (ORs) and adjusted odds ratios (AORs) were estimated to assess the strength of associations and 95% confidence intervals were used for significance tests. The proportion test was used to examine the differences in prevalence of fever among children under five and healthcare utilization for fever among children in Burkina Faso. Authors’ do their own calculations from the baseline (2013) and endline (2017) survey data for the impact evaluation of Performance-Based Financing (PBF) in Burkina Faso, to build all the table. Data were cleaned, coded and analysed using Stata software version 16.1.

### Ethical considerations

Ethical approval was obtained from the Ethics Committee of the Medical Faculty of the University of Heidelberg (Protocol number S-272/2013) and Burkina Faso National Ethics Committee (Protocol number 2013–7-06). All participants were informed about all relevant aspects of the study including its aim, procedures, potential risks and hazards. Since the subjects of this study were women aged 15–49 and children under 5 years of age, the informed consent of the women and the authorization of parents or guardians and the informed assent of the children was requested to participate in the study. The participants gave their verbal consent and decided to participate in the study voluntarily. All information would remain confidential and anonymized. No constraints or restrictions weigh on the autonomy and independence of the study or the publication of its results.

## Results

### Descriptive results of the differential prevalence of fever in children under five

Of all the children under five included in this study (1029 and 1863, respectively, in 2013 and 2017), 814 and 1,067 children, respectively, had a fever in the last four weeks preceding the survey.

Table 1 shows the distribution of children under five who had a fever in the last four weeks preceding the survey according to their household characteristics and the health practices of their mothers. Overall, the results show that, respectively, in 2013 and 2017, about eight in ten children and six in ten children (79.11% and 57.27%) had a fever in the four weeks preceding the survey.

**Table 1:** Descriptive results of differential prevalence of fever among children under five from the baseline (2013) and endline (2017) survey data for the impact evaluation of Performance-Based Financing (PBF) in Burkina Faso

Characteristics	2013			2017			Proportion test of decrease (*P*-value)
	N	Fever in 4 last week (%)	*P*-value	N	Fever in 4 last week (%)	*P*-value	
*Household size*							
1–3	333	74.47	0.024**	1,189	52.61	0.071*	p < 0.001***
4–5	480	81.04		425	54.59		*p* < 0.001***
≥ 6	216	81.94		249	59.21		*p* < 0.001***
*Number of children under five*							
1 infant	353	84.14	0.000***	678	54.69	0.325	*p* < 0.001***
2 infants	488	72.95		801	56.80		p < 0.001***
≥ 3	188	85.64		384	59.29		*p* < 0.001***
*Number of elderly persons in the household*							
No elderly	915	79.13	0.237	1,648	55.95	0.005***	*p* < 0.001***
An elderly person	89	75.28		188	68.09		0.002***
≥ 2	25	92.00		27	62.96		p < 0.001***
*Method of garbage disposal*							
In the street	510	81.76	0.022**	711	63.99	0.000***	*p* < 0.001***
Pile of filth	445	77.53		996	53.61		*p* < 0.001***
Garbage collection	74	70.27		156	50.00		*p* < 0.001***
*Mode of disposal of human excreta*							
In nature	657	81.89	0.000***	1,023	59.63	0.000***	*p* < 0.001***
Ordinary latrines	337	75.07		776	56.06		*p* < 0.001***
Improved latrines	35	65.71		64	34.38		*p* < 0.001***
*Source of drinking water*							
Unprotected sources	188	80.32	0.003***	364	60.99	0.079*	*p* < 0.001***
Protected well	99	82.83		285	52.28		*p* < 0.001***
Drilling	631	80.67		1,112	57.91		*p* < 0.001***
Household Tap	111	64.86		102	50.98		0.003***
*Children fully immunized*							
No	434	81.86	0.099*	80	55.81	0.860	*p* < 0.001***
Yes	595	77.22		1,783	57.17		*p* < 0.001***
*Received four skilled antenatal visits*							
< 4	499	79.36	0.197	643	61.74	0.005***	*p* < 0.001***
> = 4	530	78.87		1220	54.92		*p* < 0.001***
*Received postnatal care*							
No postnatal visits	315	80.32	0.938	383	60.34	0.000***	*p* < 0.001***
At least one postnatal visit	714	78.57		1,480	45.43		*p* < 0.001***
All Respondents	1,029	79.11		1,863	57.27		*p* < 0.001***

***p < .01, ** *p* < .05, * *p* < .1

This prevalence was high among children living in large households (81.94% in 2013 and 59.21% in 2017), among those living in households with three or more children (85.64% in 2013 and 59.29% in 2017) and among those living in households with at least two elderly persons (92.00% in 2013 and 62.96% in 2017).

Also noted was a particularly high prevalence among children living in households where household garbage was dumped in the street (81.76% in 2013 and 63.99% in 2017) and among those living in households that continued to defecate in nature (81.89% in 2013 and 59.63% in 2017). This prevalence was also high among children whose mothers did not attend postnatal consultations (80.32% in 2013 and 60.34 in 2017).

Whatever the year, a significant relationship was noted between variables such as household composition (household size, number of children under five, number of elderly persons), methods of garbage disposal, sources of drinking water, method of disposal of human excreta and the occurrence of fever in children under five. Regarding antenatal and postnatal care, there was no significant relationship between these variables and the occurrence of fever in children under five in 2013, but this relationship became significant in 2017.

### Descriptive results of the use of medical care for childhood fever

Table 2 shows the percentage of children under five whose fever was treated at all public or private health facilities by private doctors and community health workers. For over 75.19% and 79.76% of children with fever, respectively, in 2013 and 2017, medical health care was sought from a healthcare facility or healthcare provider. There was an increase in the percentage of children under five whose fever was treated with appropriate health care.

**Table 2:** Descriptive results of healthcare utilization for fever among child, maternal, household and health facility-level characteristics from the baseline (2013) and endline (2017) survey data for the impact evaluation of Performance-Based Financing (PBF) in Burkina Faso

Characteristics	2013		2017		Proportion test of increase (*p*-value)
	N	Sought appropriate health care (%)	N	Sought appropriate health care (%)	
*Sex of child*					
Male	442	75.31	558	80.10	0.0695*
Female	374	75.05	509	79.39	0.1267
*Child’s age*					
0–11 months	269	82.35	436	86.22	0.1651
12–23 months	335	72.10	366	77.78	0.0825*
24–35 months	213	71.00	265	71.86	0.8360
*Children full immunized*					
No	339	73.53	520	77.49	0.1845
Yes	477	79.29	547	81.40	0.3960
*Mother's age*					
15–24	333	78.38	347	78.35	0.9924
25–34	370	72.22	515	82.31	0.0003***
≥ 35	113	75.52	206	75.77	0.9603
*Mother’s marital status*					
Unmarried	21	75.00	45	78.48	0.7530
Monogamous marriage	576	76.92	664	81.10	0.0707*
Polygamous marriage	220	75.54	352	78.50	0.4105
*Mother's education level*					
No education	690	70.48	848	79.19	0.0001***
Primary education	83	75.00	141	79.76	0.4706
Secondary & +	44	87.27	78	86.03	0.8485
*Mother’s occupation*					
Not working	519	75.15	637	81.21	0.0126**
Working	290	74.66	423	78.62	0.2170
*Household size*					
1–3	263	75.90	681	79.14	0.2795
4–5	378	75.52	243	84.00	0.0116**
≥ 6	176	73.42	143	75.50	0.6722
*Number of elderly persons in the household*					
None	731	77.27	944	85.19	0.0000***
One	68	68.60	108	71.81	0.6491
≥ 2	17	75.76	15	80.58	0.7425
*Number of children under five*					
1	292	73.98	388	76.25	0.4970
2	380	75.42	459	82.65	0.0100**
≥ 3	145	77.05	220	79.95	0.5071
*Household wealth index*					
Poorest	150	71.96	198	77.97	0.1975
Poorer	150	73.02	207	78.67	0.2153
Middle	140	73.45	182	76.73	0.4987
Richer	175	75.11	214	82.57	0.0713*
Richest	203	80.47	267	81.76	0.7229
*Received four skilled antenatal visits*					
< 4	413	71.51	368	78.23	0.0311*
> = 4	403	78.88	699	80.57	0.4997
*Received postnatal visits*					
No postnatal visits	242	72.55	219	73.63	0.7941
At least one postnatal visit	574	76.31	848	81.35	0.0213*
*Waiting time in the health facility*					
Not acceptable	87	68.18	193	78.04	0.0779*
Acceptable	714	75.86	859	80.33	0.0322*
*Safety in the health facility*					
Not safe	350	69.81	434	59.73	0.0034 ***
Safe	453	81.67	633	89.66	0.0002***
*Confidence in the health facility*					
No	32	74.97	75	77.86	0.7450
Yes	771	82.50	983	79.85	0.1601
All respondents	814	75.19	1067	79.76	0.0182*

^***^p < .01, ** *p* < .05, * *p* < .1

This health care-seeking was more frequently carried out for the youngest children (less than one year) (82.35% in 2013 and 86.22% in 2017) and those living in households without elderly persons (77.27% in 2013 and 85.19% in 2017). It was also noted that children with fever tended to be deprived of medical care in the poorest households (71.96% in 2013 and 77.97% in 2017), while more than eight in ten children from the richest households benefited from appropriate care (80.47% in 2013 and 81.76% in 2017). In addition, among households with at least three children under five years old, nearly eight in ten children with fever (77.05% in 2013 and 79.95% in 2017) sought medical health care. For all these characteristics, there was an increase in the percentage of children under five whose fever was treated with appropriate health care.

It was also noticed that the frequency of the use of appropriate healthcare increased with the level of education of the mother (only 70.48% of children of mothers with no education in 2013 and 79.19% in 2017). However, 87.27% of those whose mothers had a secondary level or higher education in 2013 and 86.03% in 2017 received appropriate health care.

### Factors associated with the use of medical healthcare for fever

The results of the bivariate and multivariate logistic regression are presented in Table 3. The results of the saturated model show that being aged 12–23 months and 24–59 months, living in a very wealthy household, having within the household one elderly person and several children under five were significantly associated with seeking appropriate healthcare for childhood fever in Burkina Faso. Likewise, having a mother with a secondary or higher level education or who made at least four antenatal care visits or at least one postnatal care visit was significantly associated with seeking appropriate healthcare for childhood fever. In addition, living in an area where the health facility was safe was significantly associated with seeking appropriate care for childhood fever.

**Table 3:** Factors associated with healthcare utilization for fever from the baseline (2013) and endline (2017) survey data on the impact evaluation of Performance-Based Financing (PBF) in Burkina Faso

Characteristics	2013			2017		
	N	OR (CI 95%)	AOR (CI 95%)	N	OR (CI 95%)	AOR (CI 95%)
*Sex of child*						
Male	559	1.00	1.00	975	1.00	1.00
Female	473	0.986 (0.743–1.309)	1.014 (0.705–1.457)	888	0.957 (0.763–1.2)	0.980 (0.702–1.367)
Child’s age						
0–11 months	340	1.00	1.00	762	1.00	1.00
12–23 months	423	0.554*** (0.390–0.786)	0.459*** (0.267–0.792)	639	0.559*** (0.424–0.739)	0.357*** (0.204–0.626)
24–59 months	269	0.525*** (0.358–0.770)	0.344*** (0.182–0.649)	462	0.408*** (0.306–0.545)	0.208*** (0.115–0.376)
*Children fully immunized*						
No	429	1.00	1.00	43	1.00	1.00
Yes	603	0.726* (0.520–1.012)	1.087 (0.669–1.767)	955	0.787 (0.360–1.721)	0.703 (0.303–1.631)
*Mother's age*						
15–24	421	1.00	1.00	605	1.00	1.00
25–34	468	0.717** (0.527–0.975)	0.686* (0.445–1.057)	899	1.286* (0.993–1.666)	2.104*** (1.404–3.152)
35–49	143	0.851 (0.545–1.330)	0.677 (0.367–1.248)	359	0.864 (0.634–1.177)	1.557* (0.941–2.267)
*Mother’s marital status*						
Unmarried	26	1.00	1.00	79	1.00	1.00
Monogamous marriage	728	0.900 (0.356–1.276)	0.718 (0.239–2.140)	1159	1.177 (0.675–2.053)	1.516 (0.675–3.404)
Polygamous marriage	278	0.926 (0.357–2.401)	1.141 (0.319–4.080)	614	1.001 (0.566–1.771)	0.954 (0.401–2.267)
*Mother's education level*						
No education	872	1.00	1.00	1480	1.00	1.00
Primary education	105	0.796 (0.509–1.243)	1.151 (0.635–2.086)	247	1.035 (0.741–1.1447)	1.225*** (0.768–1.954)
Secondary & +	55	2.286** (1.019–5.126)	1.608* (0.619–4.117)	136	1.618* (0.981–2.67)	2.484* (1.103–5.594)
*Mother’s occupation*						
Not working	656	1.00	1.00	1112	1.00	1.00
Working	367	0.974 (0.726–1.308)	0.974 (0.665–1.428)	739	0.851 (0.675–1.073)	1.081 (0.771–1.517)
*Household size*						
1–3	332	1.00	1.00	1189	1.00	1.00
4–5	478	0.98 (0.707–1.358)	0.801 (0.495–1.339)	425	1.384** (1.031–1.858)	1.009 (614–1.658)
≥ 6	222	0.877 (0.594–1.295)	0.442*** (0.239–0.817)	249	0.812 (0.589–1.120)	1.273 (0.622–2.605)
*Number of elderly persons in the household*						
None	924	1.00	1.00	1648	1.00	1.00
1	86	0.699 (0.433–1.130)	0.487** (0.265–0.896)	188	0.614*** (0.437–0.863)	0.510** (0.299–0.869)
≥ 2	22	1.088 (0.397–2.982)	0.340* (0.106–1.086)	27	1.386 (0,476–4.034)	0.756 (0.189–3.020)
*Number of children under five*						
1	369	1.00	1.00	678	1.00	1.00
2	480	1.079 (0.790–1.474)	1.052** (0.641–1.726)	801	1.483*** (1.150–1.913)	1.337 (0.891–2.007)
≥ 3	183	1.181 (0.779–1.789)	1.163*(0.497–2.720)	384	1.242 (0.914–1.686)	1.857** (1.121–3.074)
*Household’s wealth index*						
Poorest	189	1.00	1.00	345	1.00	1.00
Poorer	189	1.054 (0.671–1.656)	1.570 (0.874–2.822)	361	1.042 (0.728–1.491)	1.233 (0.728–2.086)
Middle	177	1.078 (0.680–1.708)	1.439 (0.825–2.510)	318	0.932 (0.647–1.341)	1.439 (0.815–2.543)
Richer	221	1.176 (0.757–1.827)	1.229 (0.713–2.120)	373	1.339 (0.925–1.937)	1.431 (0.820–2.433)
Richest	256	1.606** (1.031–2.500)	2.014** (1.149–3.531)	466	1.266 (0.895–1.791)	2.165*** (1.223–3.834)
*Received four skilled antenatal visits*						
< 4	522	1.00	1.00	643	1.00	1.00
> = 4	510	1.488*** (1.119–1.978)	1.433** (1.006–2.041)	1220	1.154*** (0.913–1.460)	0.886 (0.625–1.256)
*Received postnatal care*						
No postnatal visits	306	1.00	1.00	383	1.00	1.00
At least one postnatal visit	726	1.219 (0.899–1.651)	0.810 (0.539–1.218)	1480	1.562*** (1.202–2.031)	1.922*** (1.329–2.778)
*Waiting time in the health facility*						
Not acceptable	110	1.00	1.00	337	1.00	1.00
Acceptable	903	1.466* (0.955–2.252)	0.751 (0.394–1.431)	1500	1.149*** (0.862–1.532)	1.436* (0.955–2.159)
*Safety in the health facility*						
No safe	573	1.00	1.00	735	1.00	1.00
Safe	459	1.959*** (1.456–2.637)	2.086*** (1.443–3.017)	1106	1.017(0.807–1.280)	1.369* (0.976–1.920)
*Confidence in the health facility*						
No	40	1.00	1.00	131	1.00	1.00
Yes	975	1.252 (0.651–2.405)	3.717* (0.834–16.562)	1717	1.127 (0.734–1.730)	1.392 (0.759–2.553)

***p < .01, ** *p* < .05, * *p* < .1

2013-Hosmer–Lemeshow test: *p*-value = 0.7541

2017-Hosmer–Lemeshow test: *p*-value = 0.9943

The mothers of children aged between 24 and 59 months are less likely to seek appropriate health care for childhood fever (AOR: 0. 344, 95% CI 0. 182–0.649 in 2013 and AOR: 0. 208, CI at 95%: 0.115–0.376 in 2017) than mothers of children between 0 and 11 months. Furthermore, mothers from the richest households were twice as likely to seek health care for childhood fever than mothers from the poorest households (AOR: 2.014, 95% CI 1.149–3.531 in 2013 and AOR: 2.165, 95% CI 1.223–3.834 in 2017). In 2013 and 2017, respectively, mothers with secondary or higher level education were 1.6 times (AOR: 1.608, 95% CI 0.619–4.117) and 2.5 times (AOR: 2.484, 95% CI 1.103–5.594) more likely to request medical healthcare for childhood fever than mothers who had no education.

## Discussion

The present study was conducted to determine the differential prevalence of fever and to identify the factors associated with appropriate health care-seeking behaviours for childhood fever in Burkina Faso.

We noted a statistically significant decrease in the prevalence of self-reported fever between 2013 and 2017. Our finding is higher than previous studies conducted in Burkina Faso [16, 33, 39, 40]. This may be due to the four-week reference period chosen to collect the data for this study as compared to the two-week reference period of other studies.

Our finding showed a statistically significant increase in health care-seeking behaviours for childhood fever between 2013 and 2017. This result could be explained by the interventions implemented in Burkina Faso since 2016. Burkina Faso implements PBF, the interventions in the field of malaria and the policy of free care for children under five and for pregnant women, for whom the financial barriers to access to health care have been removed. The study also showed that health care-seeking behaviours for childhood fever in Burkina Faso was higher than that showed in studies conducted in Nigeria [21] and Ethiopia [41]. However, it was lower than the level of health care-seeking for childhood fever in Tanzania [7] and in Gabon [42]. Because a population’s standard of living is a vital factor in health and disease [41, 43], the above inconsistent results could be due to the difference in the socio-economic status of the study participants [41, 44]. These inconsistent results could be due to the difference in the socio-economic and socio-cultural context of study participants.

The child’s age, wealth of the household, household composition, mother’s educational level, use of antenatal and postnatal care and safety in health facilities were factors significantly associated with appropriate health care-seeking behaviours for childhood fever.

In the present study, mothers were less likely to seek appropriate healthcare for children one year and older with fever. This is consistent with similar studies in Ethiopia, Tanzania and Kenya that assessed health-seeking behaviours in children under five with fever [22, 41, 45, 46]. This result could be explained by the higher vulnerability of the child under one year to malnutrition and mortality, this period being critical for children, mothers will tend to take care of their younger children, or seek appropriate health care for their younger children than their older ones [47, 48]. This finding is not consistent with study conducted in Bangladesh [49].

Mothers from the richest households were more likely to seek appropriate health care for children's fever than mothers from households with the poorest wealth status. This finding is consistent with previous studies reported in Nepal, Ethiopia, Tanzania and Nigeria, that documented financial capability as one of the determinants that strongly influences health care utilization for childhood illnesses [7, 41, 42, 50–54].

Mothers who had formal education (secondary and above) were more likely to seek appropriate health care for children with fever than mothers who had no formal education. This finding is consistent with previous studies conducted in Ethiopia, Bangladesh and Tanzania [7, 41, 50, 53]. It is known that education influences health care-seeking behaviours in communities. Educated mothers can better understand the symptoms and severity of fever and thus seek health care in a shorter period than those mothers who are less-educated.

Our finding showed that also the mothers who were using antenatal care were more likely to seek appropriate health care for childhood fever as compared to mothers who gave births at home. The possible explanation for this result might be following antenatal care enable mothers to be aware of the advantages of seeking a health care at the time of child illness.

Our study also found that children residing in households with two or more children under the age of five were more likely to receive appropriate health care for fever than children residing in households with only one child under five. In the Burkinabè context, all adult members of a household potentially have to take care of the child. This person could therefore acquire experience in caring for a child under 5 years, if there are several in this household. Therefore, a mother or guardian of a child living in a household with several children under 5 years could have this experience in terms of health care seeking. However, this finding is not consistent with another study in Tanzania [7].

Health care seeking is recognized as a complex behavioral process that is influenced by several factors [55]. In this study, we found child's age, wealth of the household, household composition, mother’s educational level, use of antenatal and postnatal care and safety in health facilities were significantly associated with appropriate health care-seeking behaviours for childhood fever. Based on the results of this study, concentrated efforts aimed at sensitizing the population (especially women of childbearing age) to improve sanitation and to the use of family planning (household composition), skilled antenatal care and postnatal care will help reduce the prevalence of fever in children under five and improve the use of medical healthcare for childhood fever. This indicates that activities aimed at increasing knowledge and awareness of the importance of family planning, skilled antenatal care, sanitation, and other preventive measures for childhood fever should be conducted with women of childbearing age.

Our study has some limitations. As the study was cross-sectional and data were collected in a short period, we could not capture changes in the health care-seeking behaviours of mothers between seasons. Moreover, in this study, we did not analyse the health care-seeking behaviours of mothers in terms of the duration and severity of the fever due to the fact that this study did not collect data on the severity and duration of fever. Also, due to the fact that the data we used are cross-sectional and not experimental, causality could not be established. Furthermore, although our study’s focus was a sample of randomly selected households in the regions, the generalizability of our results may be limited given that study regions and districts of the study were purposely selected. The construction of the results in this study depended on women's self-report of childhood fever and care-seeking behaviours. Perception and accurate recall of fever in young children pose many problems of reliability and validity. The accurate recall of symptoms depends largely on the respondent's knowledge of these symptoms and the subjective perception of the disease by the respondent and / or other household members. Despite these limitations, the strength of our study is evident. This is a large cross-sectional and population-based study that covers six out of thirteen regions of the country, with a large sample. However, to reduce as much as possible the various limitations and possible biases of this study, descriptive and multivariate analysis methods were combined.

## Conclusions

This study found that the occurrence of fever in children under five was associated with variables relating to household composition and sanitation and those relating to the mothers' use of antenatal and postnatal care. This study also found that important factors related to the individual household composition and health facility are associated with seeking appropriate health care for childhood fever in Burkina Faso. These findings underscore the need for interventions that would improve mothers' pursuit of appropriate medical care for their children. These interventions should take into account individual, household and health facility characteristics and could include, but are not limited to, increasing girls' education; improving household living conditions, including sanitation; improving the level of use of prenatal and postnatal care; and improving the quality of care as well as safety in health structures. Hence, there is a need that the health workers and program planners design a tailored health message by for mothers about childhood fever and appropriate health care seeking for children with fever. Further studies should focus on longitudinal and experimental exploration.

## Data Availability

The datasets used and/or analysed during this study are available from the corresponding authors on reasonable request.

## Abbreviations

DHS: Demographic and health surveys
INSD: National institute of statistics and demography
PBF: Performance-based financing
